# Pupil Cycle Time Distinguishes Migraineurs From Subjects Without Headache

**DOI:** 10.3389/fneur.2019.00478

**Published:** 2019-05-08

**Authors:** Melissa M. Cortez, Natalie Rae, Leah Millsap, Nick McKean, K. C. Brennan

**Affiliations:** ^1^Department of Neurology, University of Utah, Salt Lake City, UT, United States; ^2^School of Medicine, University of Utah, Salt Lake City, UT, United States

**Keywords:** migraine, pupil cycle time, craniofacial autonomic symptoms, central sensititization, trigeminal sensitization

## Abstract

Migraine is a neurological disorder characterized by paroxysms of head pain accompanied by trigeminovascular system activation and autonomic dysfunction. Diagnosis is currently based on clinical diagnostic criteria. Though physiological differences exist between migraineurs and non-headache controls, true physiological biomarkers have been elusive, especially for the full clinical spectrum of migraine, inclusive of chronic, episodic, and probable migraine. We used edge-light pupil cycle time (PCT) as a probe of the pupillary light circuit in migraine, paired with clinical assessment of migraine characteristics, and compared these to non-headache controls. We found significantly increased PCT in probable, episodic, and chronic migraine, compared to controls. Additionally, increased PCT correlated with the presence of craniofacial autonomic symptoms, linking pupillary circuit dysfunction to peripheral trigeminal sensitization. The sensitivity of PCT, especially for all severities of disease, distinguishes it from other physiological phenotypes, which may make it useful as a potential biomarker.

## Introduction

Migraine is a common, recurrent headache disorder characterized by paroxysms of head pain accompanied by trigeminovascular system activation and autonomic dysfunction. Diagnosis is currently based on clinical diagnostic criteria and may be diagnostically categorized as probable, episodic, or chronic migraine, based on duration, number of attacks, and associated symptoms. Thus, far while clinical signs of migraine chronification (previously termed “transformation”) and central sensitization are now recognized, such as cutaneous allodynia (1, 2), few human studies have shown abnormalities in *physiology* present across the full clinical spectrum of migraine inclusive of chronic, episodic, and especially probable migraine. In fact, to our knowledge, no physiologic test has yet been demonstrated to separate out probable migraine (PM) from non-migraineurs—a diagnostic category most practitioners consider to be clinically actionable.

Changes in pupillary function have been variably observed during the migraine headache attack (3–5), as well as inter-ictally (5, 6). Recent evidence supports the possibility of a disease gradient in the expression of pupillary responses to light, perhaps linked to the presence of photophobia (7), a well-recognized symptom of central sensitization most evident in chronic migraine (CM). Craniofacial autonomic signs and symptoms are now recognized to be relatively common in migraineurs (37–73%) and often co-occur with photophobia and allodynia (8, 9). These facial signs and symptoms are thought to arise from peripheral nociceptive activation of the trigeminal-autonomic reflex, leading to efferent activation of cranial nerves targeting the nasal mucosa, lacrimal glands, other facial structures (10). Alterations in the pupillary light reflex (PLR), in the setting of central sensitization, may occur by similar mechanisms (11), though the relationship between pupillary function and craniofacial autonomic symptoms (CAS) has not been directly explored.

Edge-light pupil cycle time (PCT) was initially developed by Miller and Thompson in 1978 as a test of optic nerve afferent pathway disease (12). Soon after, the technique was extended for use as a screen of the pupil's entire light reflex arc, inclusive of efferent pupillary pathways (13). Pupil cycle time has shown sensitivity for both parasympathetic (14) and sympathetic (15) disorders of the PLR. Thus, we deployed PCT for the assessment of the pupillary light circuit in migraine, paired with clinical assessment of migraine characteristics.

## Materials and Methods

### Participants

In total, 98 subjects (31 male/67 female) aged 15–75 years were recruited into two groups: (1) migraine headache, and (2) age and sex-matched non-headache (NH) controls. Subjects were recruited between June 2015 and September 2018, from local community and the University Neurology Headache and General Neurology clinics, as well as community volunteers via word of mouth, internet, and flier advertisements. Institutional Review Board approval was obtained from the University of Utah Human Studies Committee (IRB_00085309 and IRB_00064447). All participants completed written informed consent; for those under the age of 18, participant assent, paired with parental (or legal guardian) informed consent and permission, were obtained. Headache diagnosis was based on 2013 International Classification of Headache Disorders III-beta criteria (16). Upon completion of a structured clinical questionnaire (described further below), the migraine group was further divided into episodic migraine (EM), chronic migraine (CM), and probable migraine (PM) for a total of 73 headache subjects (28 migraine with aura, 45 migraine without aura).

Episodic and probable migraine participants were studied after being headache-free for at least 48 h, and subjects were excluded if a migraine occurred within 24 h of testing. Chronic migraine subjects were studied when migraine attack-free for at least 48 h, though testing during daily or non-migrainous headaches was permitted. Subjects had not used opiate medication or migraine-specific abortive medications during the 48 h prior to testing. Headache diaries were used to assess attack frequency for 1 week prior to testing and for subsequent attacks occurring within the 2 weeks of testing, as well as medication use. Subjects did not take medications (including eye drops other than artificial tears, or prophylactic treatment for migraine, including psychotropics, antihistamines, benzodiazepines, barbiturates, and derivatives), and denied a history of comorbid medical, ocular, or neurological disorder (e.g., prior eye injury, idiopathic blepharospasm, optic nerve disorder), that is known to directly affect autonomic function or pupillary control (including diabetes). Subjects were instructed not to consume alcohol, caffeine or nicotine for at least 4 h prior to testing. The group of age and sex matched control subjects reported no history of headache and were studied in their usual state of health.

### Measurements

#### Questionnaire

Subjects completed a modified written Structured Migraine Interview (17) along with a headache diary to characterize migraine diagnosis and headache frequency. The Migraine Disability Assessment (MIDAS) (18), Headache Impact Test (HIT-6) (19), Fatigue Severity Scale (FSS) (20), Patient Health Questionnaire (PHQ-9) (21, 22) and Generalized Anxiety Disorder (GAD-7) (23, 24) were also collected to assess headache impact and related disability, as well as fatigue and affective symptoms. As there are no currently available, validated tools for headache-associated cranial autonomic symptoms (CAS), we based our assessment on those used by Gelfand et al. (25) and the ICHD-III proposed definition of CAS (26). Subjects replied “yes” or “no” to the following eight symptoms associated with their usual headache: conjunctival injection or lacrimation, nasal congestion or rhinorrhea, eyelid swelling, forehead/facial sweating, forehead/facial flushing, changes in pupil size, droopy eyelid, sense of fullness in the ear.

#### Edge-Light Pupil Cycle Time (PCT)

Edge-light PCT was assessed using methods adapted from Miller and Thompson (12) as a measure of the relative integrity of both afferent and efferent pupillary pathways. This technique differs from other types of pupillary oscillations (27), which tend to be of irregular cycling duration, including that of pupillary unrest (e.g., hippus) (28), which occurs under diffuse illumination, and from the significantly slower, rather episodic, pupillary oscillations that occur under dark conditions in the fatigued or drowsy subject (29–32). In contrast, edge-light PCT uses a directed, narrow beam of light at the pupil edge, which exploits the normal pupil's light reflex arc and produces a fairly brisk, and regular oscillation. Pupil cycle time has been shown to be stable across repeated testing and is not significantly affected by refractive error, sex, or iris color (12, 13). Thus, we selected this assessment as a simple method for assessing overall pupil responsiveness to light across groups.

**Figure 1:**
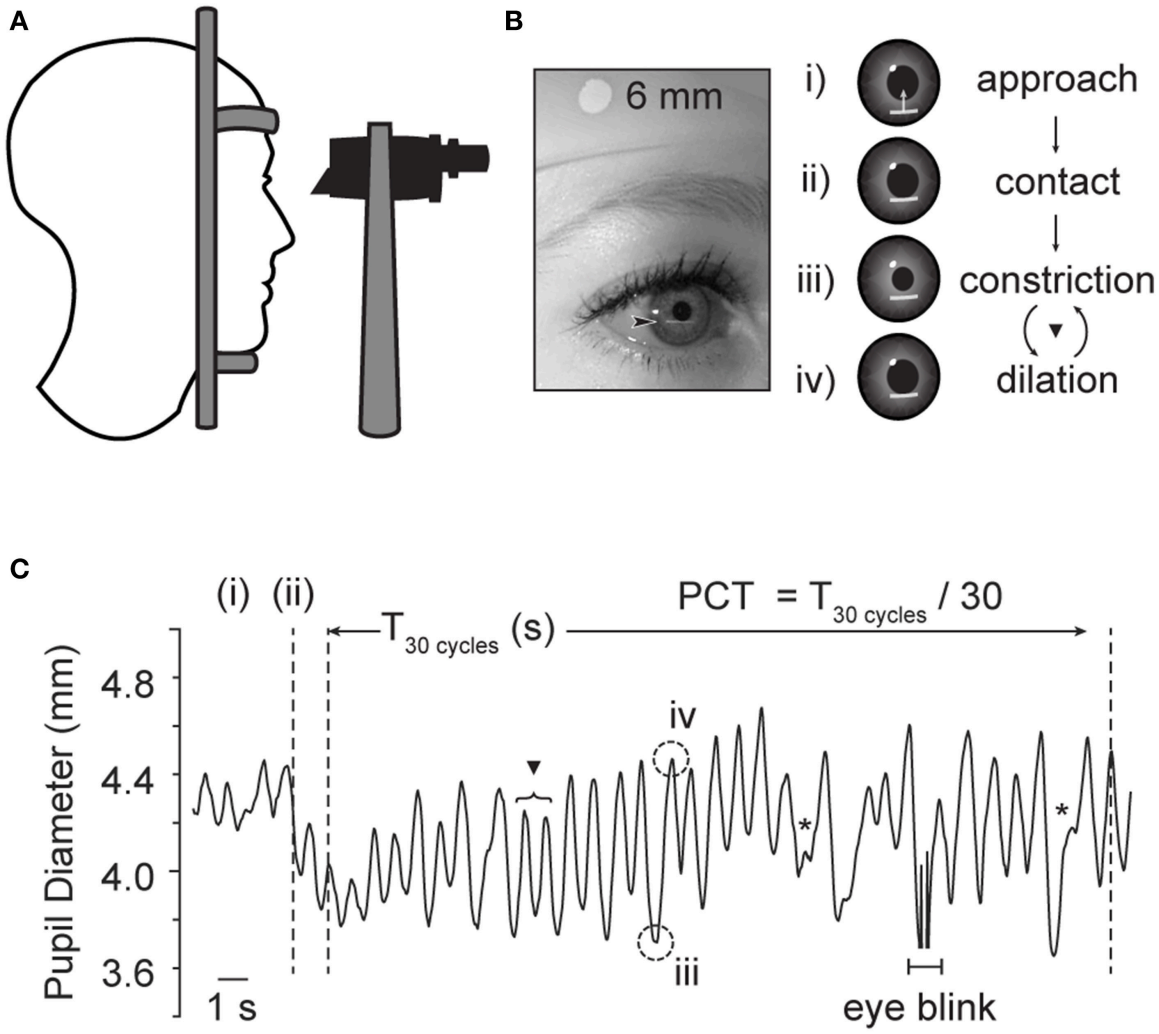
Examination technique for edge-light pupil cycle time (PCT). **(A)** The subject is comfortably seated in front of a slit lamp, and the pupil visualized through the microscope. **(B)** A horizontal slit beam of light is positioned inferior to the plane of the iris, and elevated until contact is made with the pupillary margin, initiating constriction. The beam is held in place, so that the pupillary constriction brings the pupillary margin out of contact with the light. Once out of the light, the pupil then spontaneously re-dilates, eventually returning the edge of the pupil to contact the light beam once again. Thereby, this method produces a sustained oscillation between dilated and constricted states **(C)**. One cycle is the time it takes for a complete loop of the pupil reflex arc to be completed. ▾ Denotes full cycle counted by PCT method. *Denoted superimposed beats of pupillary unrest, which are not thought to interfere in the oscillating rhythm of interest (12). Plot based on infrared pupil recording during routine PCT collection in a healthy subject, sampling rate 30 Hz. Acknowledgments: Jeremy Theriot for illustration.

The examination set-up, and a representative example of pupil diameter change over time with this technique is shown in Figure 1. In this test, the subject is seated at a slit-lamp in a dimly lit room (<1 lux) and asked to gaze toward a designated object consistent with the subject's far point. After a 3-min dark adaptation period, a horizontally oriented beam of light (5 mm wide, 0.5 mm thick) is positioned just below the inferior aspect of the pupillary margin, and slowly elevated until it contacts the inferior edge of the pupil. The intensity of the light beam, range 10–100 lux, was kept at the lowest intensity necessary to produce pupil constriction, in order to maximize subject comfort for the duration of the test. In normal subjects, the light beam induces brisk pupillary constriction, moving the pupillary margin outside of the light stimulus; the pupil then spontaneously re-dilates until encountering the beam once again, creating a cycle. Here, the examiner maintains the location of the light beam in order that the iris remains outside the light while constricted, but re-contacts the light upon re-dilation. One cycle is counted as one pupillary constriction, followed by re-dilation; each cycle is observed and counted through the binocular scope of the slit lamp. According to previously published methods, after regular cycling is established (typically 2–3 oscillations), a total of 100 cycles, divided into 3 trials of 30, 30, and 40 cycles each, are directly visualized and counted by the examiner; cycle time is then reported in milliseconds/cycle (msec/cycle) (12, 14). In order to minimize learning curve and the risk of systematic biases, all examiners were trained by the same experienced examiner, blinded to study group, and used a standardized script for instructions to the study subject.

For analysis purposes, the overall pupil cycle time for each eye was determined by averaging the three trials for each eye. Prior authors have noted occasional irregular beats of constriction, superimposed on the regular oscillations of the edge-light based cycling; these “mini-fluctuations” are thought to represent superimposed “pupillary unrest,” and thus examiners were trained not to count these (12). Cycle time was not identical between sides, though there were no significant differences in PCT between right vs. left eyes in either the NH nor the Migraine-All group (Wilcoxon signed-Rank test, *p* = 0.68 and 0.80, respectively). For the purposes of analysis, we used the longest of the two sides, according to previously published methods (14). All experiments were performed in a quiet, controlled environment, to limited external sources of excitation.

#### Baseline Pupil Size

Baseline pupil size data was obtained in a separate protocol, performed during the same testing session, prior to PCT data collection. Here, dark-adapted pupil size was obtained after a 10 min acclimation period to the testing environment, and an additional 1 min of dark adaptation using a binocular pupillometer (DP-2000, Neuroptics Inc, Irvine, CA; image acquisition 30 Hz, pixel resolution 0.05 mm).

#### Statistical Methods

Visual inspection of the data, followed by Shapiro-Wilk normality test, was applied to each parameter to assess for distribution of data. Overall, our test parameters were not normally distributed, thus Kruskal-Wallis was utilized for across group comparisons, and Wilcoxon rank sum test was used for *post-hoc* pair-wise comparisons. In these instances, *p*-values were considered significant only following Bonferroni correction for multiple comparisons. Correlations were made initially using two-tailed Spearman's correlation as a conservative method, and confirmed with age-adjusted partial correlations with Holm's correction for multiple comparisons. Multiple regression analysis, using standard least squares method with Box-Cox transformation as indicated, was performed to evaluate effect of the following covariates: PCT with anxiety (GAD) + depression (PHQ) + fatigue severity (FSS), and PCT with baseline pupil + age. A Bland-Altman assessment for inter-rater agreement was used to compare PCT calculations between two raters for a selected subset of data (n = 17). Finally, measurement dispersion between trials 1 and 3 of PCT within the control and migraine groups was assessed using quartile coefficient of variation. Results were considered significant for *p*–values ≤ 0.05, except where Bonferroni was applied. Statistical analyses were performed with R for Windows (Version 3.5.1; R Core Team, Vienna, Austria) and JMP version 14.2.0 (2019, Windows).

## Results

Baseline characteristics and headache-specific clinical characteristics are summarized in Tables 1, 2, respectively. There were no significant differences in age or sex-distribution between the NH and migraine groups. Migraine subjects reported significantly more depression (PHQ-9), anxiety (GAD-7), and fatigue (FSS) related symptoms than NH controls. Age of headache onset did not significantly differ between migraine subgroups. As expected, based on diagnostic criteria, headache days per month was significantly higher in CM subjects; similarly, MIDAS and HIT-6 scores were significantly higher in CM than EM and PM.

**Table 1:** Clinical characteristics.

	**Non-headache control**	**Migraine-all**	***p*-value**
*n*	25	73	
Sex (%F)	60%	71%	0.18
Years of age (median, min–max)	27, 17–52	28, 15–75	0.17
FSS (median, min–max)	2.2, 1.0–4.1	3.6, 1.2–6.4	<0.0001
PHQ-9 (median, min–max)	2, 0–10	5, 0–23	<0.0001
GAD-7 (median, min–max)	0, 0–7	4, 0–21	<0.0001

*F, female; FSS, Fatigue Severity Score; GAD, Generalized Anxiety Disorder; max, maximum; min, minimum; PHQ, Patient Health Questionnaire*.

*Sex: chi-square test*.

*Age, Fatigue Severity, PHQ, GAD: Wilcoxon rank sum test*.

**Table 2:** Migraine group clinical characteristics.

	**PM**	**EM**	**CM**	***p*-value**
Age of HA onset, years of age (median, min–max)	15, 6–41	14, 5–42	16, 3–52	0.37
HA days per month (median, min–max)	5, 0–10	5, 1–27	20, 10–30	<0.0001
MIDAS (median, min–max)	5, 0–42	6, 0–62	48, 0–78	0.0001
HIT-6 (median, min–max)	50, 9.5–70	57, 40–68	63, 52–72	0.002
HA-associated CAS, one or more out of 8 (%)	36%	40%	48%	0.54
HA-associated CAS, total number reported out of 8 (median, range)	1, 0–3	1, 0–4	2, 0–5	

*CAS, craniofacial autonomic symptoms; CM, chronic migraine; EM, episodic migraine; HA, headache; HIT, Headache Impact Test; max, maximum; MIDAS, Migraine Disability Assessment; min, minimum; PM, probable migraine*.

*Age, HA days, MIDAS, HIT, CAS: Wilcoxon rank sum test*.

### Pupil Cycle Time (PCT)

**Figure 2:**
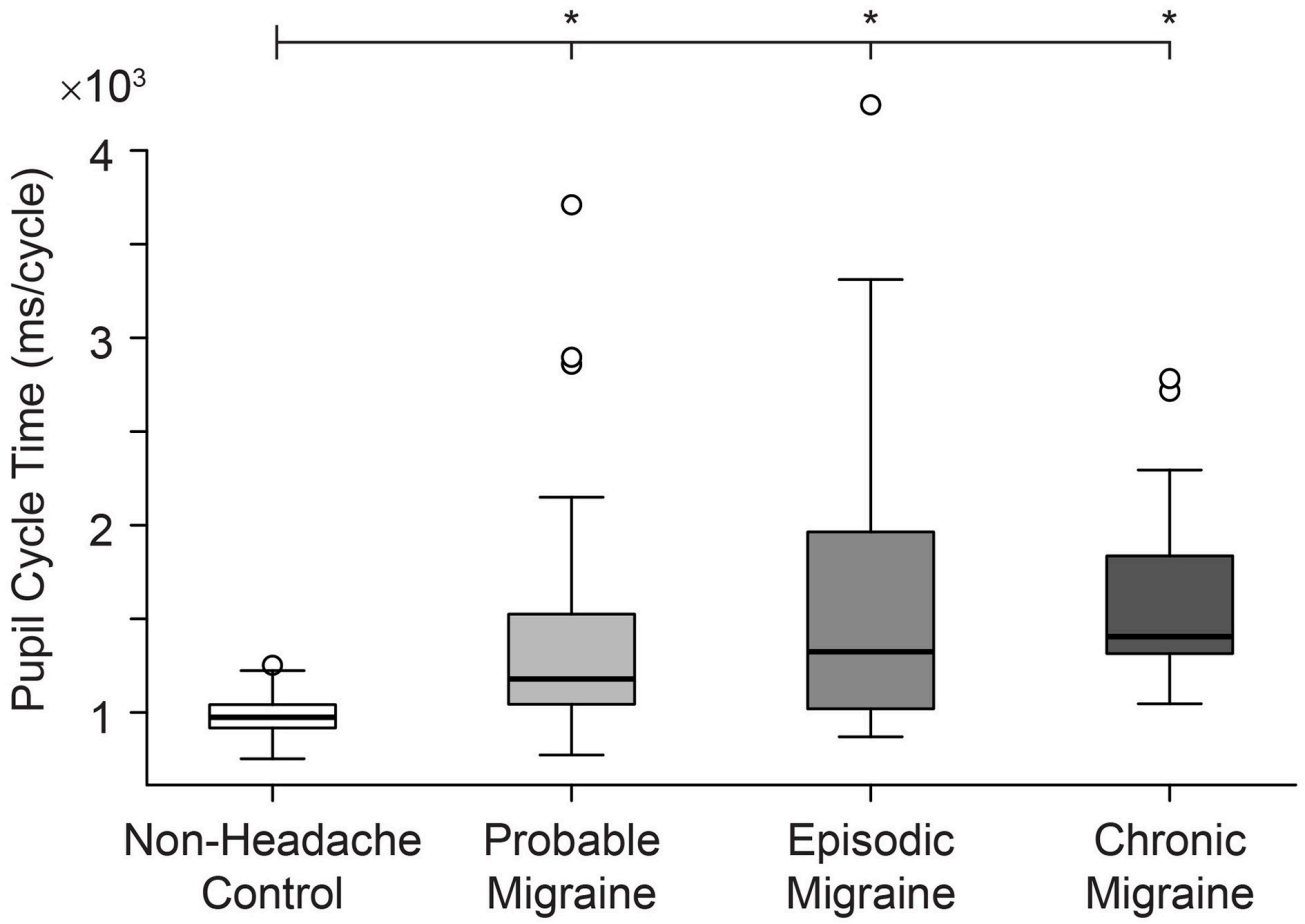
Pupil cycle time (PCT) differs significantly in all migraine groups compared to non-headache controls.

Longest pupil cycle time was significantly different across all groups (Kruskal-Wallis rank sum test, *p* = 0.00001) (Figure 2). Pair-wise comparisons (2-sample Wilcoxon test) confirmed a significantly longer PCT in PM vs. NH (*p* = 0.0005), EM vs. NH (*p* = 0.001), and CM vs. NH (*p* < 0.0001). While there were no significant differences across migraine sub-groups (Kruskal-Wallis rank sum test, *p* = 0.38), median values appear to show a gradient between NH<PM<EM<CM. See Table 3 for data summary.

**Table 3:** Pupil testing.

**Subject groups**	**Non-headache control**	**Migraine-all**	**PM**	**EM**	**CM**
Baseline pupil size, mm (Median, min–max)	6.6, 5.5–9.2	6.6, 2.7–8.1	6.6, 3.1–8.1	6.6, 5.3–7.5	6.2, 2.7–8.0
PCT, msec (Median, min–max)	1034.4, 800.8–1251.9	1353.3^*^, 892.1–4244.4	1273.3^*^, 892.1–3711.00	1353.3^*^, 895.4–4244.4	1440.1^*^, 1210.8–2782.9

*CM, chronic migraine; EM, episodic migraine; max, maximum; mm, millimeter; min, minimum; msec, millisecond; PM, probable migraine; PCT, pupil cycle time*.

* *Significant difference from control*.

The significant difference seen between the NH vs. Migraine-all groups remained significant after Box-Cox transformation and least squares multiple regression analysis controlling for GAD + PHQ + FSS (*p* = 0.03); subgroup comparisons maintained significance or trended toward significance (NH vs. PM, *p* = 0.1; NH vs. EM, *p* = 0.1; NH vs. CM, *p* = 0.0009). There were no significant differences in baseline pupil sizes across NH and migraine groups (*p* = 0.99). PCT remained significantly longer in the migraine groups after controlling for dark-adapted baseline pupil diameter and age (NH vs. Migraine-All, *p* = 0.02; NH vs. PM, *p* = 0.01; NH vs. EM, *p* = 0.1; NH vs. CM, *p* = 0002).

One or more headache-associated CAS were reported in 29 of 73 (40%) of headache subjects overall; of these, CAS were most commonly reported in CM (48%), followed by EM (40%) and PM (36%). Pupil cycle time significantly correlated with number of CAS in the Migraine-All group (Spearman rho 0.40, *p* = 0.04). Within the migraine group (n = 73), 19% reported at least one unilateral CAS, which corresponded with their typical headache side; 39% reported alternating CAS; and 42% reported bilateral CAS. Forty-four of the 73 headache subjects (60%) reported habitual lateralization of headache location; within this group, there was no significant difference between right and left PCT (paired-sample Wilcoxon test, *p* = 0.24), nor were there significant differences between PCT in headache subjects who reported alternating, lateralizing or non-lateralizing headaches (Kruskal-Wallis rank sum test, *p* = 0.34). Finally, PCT did not differ significantly in those who reported unilateral CAS, compared to their non-symptomatic side.

### Inter-rater Agreement and Measurement Dispersion of Pupil Cycle Time

In our study, there were no significant differences in PCT between examiner groups of NH (*p* = 0.23) and migraine (*p* = 0.25). Additionally, Bland-Altman assessment for agreement between two raters on a subset of data (*n* = 17) indicated that the 95% limits of agreement between the two methods ranged from of −36.83–796.09, with a correlation coefficient of *r* = 0.79.

Measurement dispersion was similar between trials 1 and 3 of PCT in both NH and Migraine-All, though more variability was noted in the Migraine group overall: quartile coefficients of variation of 0.11 and 0.10 (NH), and 0.23 and 0.28 (Migraine-All), respectively.

## Discussion

Our study provides insight into the integrated pupillary response to light in migraine headache, and links this network output to craniofacial autonomic signs and symptoms. We show that PCT is significantly prolonged across all migraine subjects, with an apparent disease gradient of PCT prolongation across clinical subgroups (CM>EM>PM>NH; Figure 2). Our data show that migraine subjects, including those with PM under ICHD-III-beta definitions, can be distinguished from healthy NH controls, not only on the grounds of symptomatic profile, but also physiological measures.

Furthermore, we found a significant correlation between PCT and CAS, with increasing PCT in those with the greatest number of craniofacial autonomic symptoms, which also exhibit the same disease gradient (Table 2). While, peripheral trigeminal sensitization in migraine is thought to be a prerequisite for the head pain itself (10, 33), headache attack-associated CAS may reflect a more marked or prolonged underlying peripheral trigeminovascular sensitization, and are associated with a higher disease burden (9). Interestingly, those with unilateral CAS have been noted to benefit more from 5-HT-1B/1D agonist-based treatments than those without CAS (34). Further study is needed to understand whether PCT might be a useful objective tool to study treatment responsiveness.

### Relationship of Pupillary and Cranial Autonomic Symptoms to Peripheral and Central Sensitization in Migraine

Patients with co-existing allodynia and photophobia—both well-recognized signs of central sensitization—are also more likely to report headache-associated CAS (9), implicating CAS in the process of central sensitization as well as peripheral trigeminal sensitization (10). Thus, far while multiple *clinical signs* of migraine chronification and central sensitization are now measurable through quantitative sensory testing and questionnaires, few studies have shown abnormalities in objective physiology present across the full clinical spectrum of migraine inclusive of chronic, episodic, and in particular, probable migraine. We show data linking pupillary responses to light in migraine, to signs of peripheral trigeminal and central sensitization, with longer PCT correlating with increased frequency of headache-associated craniofacial autonomic signs and symptoms. This finding builds on recent findings showing altered pupillary light responses in migraineurs with the lowest light sensitivity thresholds (7). Future investigation into the relationship of light sensitivity, allodynia, and quantitative pupillary responses to light will aid in parsing these relationships.

While CAS can correspond with habitual headache side (19% in our study), a majority of adult migraineurs report bilateral or alternating CAS (81% in our study; 67–95% in Lai et al.) (35). Based on this, it is not surprising that we did not see significant relationships between CAS laterality and PCT asymmetry. Further, this may provide preliminary data to support the possibility that PCT reflects underlying circuit dysfunction, which is not necessarily lateralizing. Though, given the relatively small proportion of our sample with unilateral CAS, we were likely underpowered to detect significant relationships between strictly lateralizing CAS and PCT.

### Implications to Our Understanding of Migraine Headache

In this study, we utilized edge-light PCT, which was originally developed as a measure of the relative integrity of both afferent and efferent pupillary pathways, and thus a measure of the integrated pupillary circuit response. The first applications of this method were in afferent disorders (retinal and optic nerve dysfunction) (36–38), though it fell out of favor for this use with the development of more specific methods for optic nerve assessment (39). Subsequent studies have supported use of PCT for evaluation of both parasympathetic and sympathetic lesions of the PLR reflex arc. Martyn and Ewing (14) applied the technique in subjects with diabetic autonomic neuropathy, where PLR correlated with abnormal cardiovascular autonomic function, and was pharmacologically localized to the efferent (parasympathetic) limb of the PLR arc (14). Blumen et al. applied PCT in subjects with unilateral Horner's syndrome, and showed prolongation in central, preganglionic, and postganglionic sympathetic lesions (15). In our study, in the absence of adjunct pharmacological or quantitative PLR testing, we are not able to make conclusions regarding localization of underlying parasympathetic vs. sympathetic dysfunction, though our data could be seen as consistent with prior observations of mixed sympathetic and parasympathetic hypofunction in migraineurs in the inter-ictal phase (6, 7, 40, 41). Though the literature is mixed, and the majority show relatively subtle differences and/or variable patterns between groups (5, 42).

Beyond sympathetic and parasympathetic localization, we favor the interpretation of PCT as a sensitive (but not completely specific) indicator of whole pupillary circuit (dys)function, with the potential to detect, or even amplify, subtle changes in pupillary light responses, including those of central origin (43), which are of particular interest in migraine where cortical processing and central sensitization are implicated (10). Foundational studies, featured in a historical review by Lowenstein and Loewenfeld (29), highlighted the role of not only brainstem mediated sympathetic and parasympathetic influences on maintenance of the PLR arc, but also importantly, cortical influences. More contemporary methods have explored the role of central control of autonomic outflow to the iris with particular attention to spontaneous oscillations of pupil size under both light and dark conditions (27, 43, 44); premotor autonomic nuclei, including the paraventricular nucleus of the hypothalamus, and the dorsal raphe nucleus and locus coeruleus of the midbrain, are light sensitive and are of particular interest when considering centrally mediated responses to light.

Current understanding of migraine pathophysiology implicates multiple common neuroanatomical sites within the PLR arc (10, 44): cortical and hypothalamic projections provide descending modulation via the periaqueductal gray (PAG), nucleus cuneiformis (NCF), and rostroventromedial medulla (RVM), which have also been implicated in models of pain sensitization; direct projections from the paraventricular nucleus (PVN) of the hypothalamus to the trigeminal nucleus caudalis (TNC) are implicated sites in migraine models. The PAG and PVN are both involved in the classically recognized “light-inhibited” sympathetic pupillary pathways, where light causes a sympatho-inhibitory effect. As the circuit mechanisms of migraine remain poorly understood, we can only speculate at this point on the ultimate source of migraine-associated dysfunction; however it is likely that, as with other disorders of circuit function (rather than, for example, “lesion”-based disorders like stroke or multiple sclerosis) the phenotype arises from altered synaptic weighting within the circuit, rather than the destruction or explicit dysfunction of any one circuit element (10, 44).

## Limitations

In our study, the range of PCT observed in normal individuals was broader than previously reported normal control groups, where the reported normal upper limit was 935–946 (36). This highlights one of the pitfalls of PCT, in that it is observer dependent, and may be artificially prolonged by undetected cycles, interruption by frequent blinking, or need to follow a moving pupil in cases of eye movement (45, 46). To address this, the first 50 subjects were collected by a single investigator (9 NH and 41 migraineurs), with the second half (48 total; 16 NH and 32 migraineurs) of our sample performed by two other individuals trained by the original investigator. In our study, inter-rater variability appeared minimal (as above in Results, Inter-rater agreement and measurement dispersion of pupil cycle time section), though in working with this technique, it is evident that the method would benefit from updating, including use of currently available dynamic pupillometry and objectively defined cycle counting parameters.

As with most complex physiological mechanisms, the broad array of involved structures complicates interpretation. We have attempted to address this through careful screening of medical history for confounding ocular or central nervous system disorders. It is also well-recognized that differences in fixation and stimulus luminance can result in variable changes in amplitude and latency of pupil contraction (47), which could alter PCT. Thus, our protocol included a strict point of visual fixation to decrease eye movement and fixation-based pupillary changes, a standardized light stimulus, and trials where blinking or eye movement disrupted reliable recording of PCT were discarded (4 trials total across all subjects). Additionally, some might mistake PCT for measurement of pupillary “unrest” (aka hippus), for which the underlying mechanisms are unknown. However, as discussed above, hippus is inherently irregular with variable amplitude, and is present in diffuse (rather than a focused beam) illumination (12), which were importantly not the characteristics of the edge-light PCT elicited by our protocol.

## Conclusions

Our data shows that migraine subjects, including for the first time those with probable migraine, can be distinguished from NH controls not only on the grounds of symptomatic profile, but also on physiological measures. Furthermore, we show data linking pupillary responses to light to signs of peripheral trigeminal and central sensitization, with increases in PCT correlating with increased craniofacial autonomic signs and symptoms. Finally, while PCT does not have circuit localizing function without pharmacological manipulation, we have shown data revealing significant differences in pupillary physiology between non-headache controls, and migraineurs—inclusive of probable migraine. Given that PCT is relatively simple, and could be amenable to automation and standardization of methodology, such a tool could be used to detect the earliest phases of peripheral trigeminal sensitization, potentially identifying opportunities for early intervention, as emerging “disease modifying” therapies in migraine are deployed.

## Ethics Statement

This study was carried out in accordance with the recommendations of The University of Utah Institutional Review Board (IRB) with written informed consent from all subjects. All subjects gave written informed consent in accordance with the Declaration of Helsinki. The protocol was approved by the University of Utah IRB. This study did not involve vulnerable populations.

## Author Contributions

MC, NR, and KB conceived of and planned the experiments, as well as conducted recruitment. NR performed the measurements and data collection, MC supervised the data collection. MC, LM, and NM processed the experimental data and performed analysis. KB aided in interpreting the results. MC drafted the first draft of the manuscript and drafted the figures. NR wrote sections of the manuscript. KB wrote sections and edited the manuscript. LM and NM reviewed and edited the manuscript. All authors take responsibility for the contents of the manuscript.

### Conflict of Interest Statement

The authors declare that the research was conducted in the absence of any commercial or financial relationships that could be construed as a potential conflict of interest.
